# Facility upgrade for superheavy-element research at RIKEN

**DOI:** 10.1140/epja/s10050-022-00888-3

**Published:** 2022-12-09

**Authors:** Hideyuki Sakai, Hiromitsu Haba, Kouji Morimoto, Naruhiko Sakamoto

**Affiliations:** RIKEN Nishina Center, 2-1, Hirosawa, Wako, 351-0198 Saitama, Japan

## Abstract

The RIKEN Nishina Center (RNC) executed an accelerator upgrade project for the heavy-ion linac (called RILAC). A superconducting RIKEN linear accelerator (SRILAC) and a new superconducting electron-cyclotron-resonance ion source (SC-ECRIS) to boost the final energy and intensity were constructed, aimed at synthesizing a new superheavy element, 119, through a hot fusion reaction. The project included the construction of a gas-filled recoil ion separator (GARIS-III) suitable for detecting the residues of the hot-fusion reaction. To avoid research interruption during the SRILAC construction period (2017–2019) and gain experience in hot-fusion reaction processes, GARIS-II located in the GARIS experimental hall in LINAC building was moved to the E6 experimental hall in Nishina building. Certain exploratory measurements were performed employing the beams accelerated by RILAC2 and the RIKEN ring cyclotron (RRC), which is a part of the existing accelerator complex of the radioactive isotope beam factory (RIBF). Further, commissioning experiments with the upgraded facility (SRILAC and GARIS-III) were performed. The upgrade project and its commissioning results are chronologically described in this article.

## Introduction

**Fig. 1:**
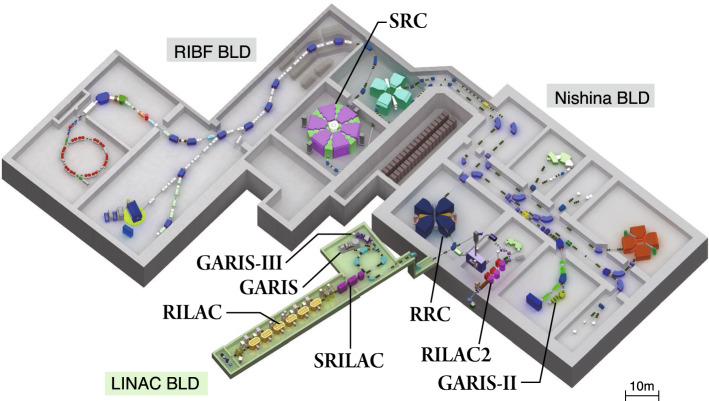
Aerial-view of the present RNC facility (orthographic projection) consisting of three buildings (LINAC, Nishina, and RIBF) due to historical reasons. RILAC, SRILAC, and GARIS-III are in LINAC building, whereas RILAC2, RRC, and GARIS-II are in Nishina building

RIKEN has a long nuclear-science research tradition pioneered by Yoshio Nishina [1] who constructed the first cyclotron (26-inch) in 1937 outside the United States (second in the world). In 1975, the RIKEN linear accelerator (RILAC) was built to extend the research activities by providing ions heavier than Ne [2]. RILAC was followed by a series of cyclotron constructions constituting an accelerator facility capable of providing U-ions up to 345 MeV/*u*. The RIKEN Nishina center (RNC) was established in 2006 to operate/manage the accelerator complex. The Bird’s eye figure of the present RNC facility where research using various heavy-ion beams is being actively pursued is shown in Fig. 1.

One of the recent epoch-making achievements of the RNC is the discovery of element nihonium [3–5]. Nihonium was synthesized through the cold fusion reaction $^{209}$Bi$+^{70}$Zn$\rightarrow ^{278}$Nh$+n$. A $^{70}$Zn beam with E=5.04 MeV/*u* was provided by RILAC. The evaporation residue, $^{278}$Nh, was separated using a gas-filled recoil ion separator (GARIS) [6, 7] and identified based its $\alpha$ decay chain.

In 2016, the RNC commenced a new comprehensive superheavy element (SHE) research program, abbreviated here as the “SHE project” for convenience. Its main objective was to expand the periodic table of elements by synthesizing new superheavy elements. After the discovery of oganesson (Z = 118) [8], the aim of the SHE project was to discover an element beyond Z$$=118$$. Considering the possible fusion reaction between the beam of a stable isotope and an actinoid target, which is easy to manipulate in terms of the radiation safety and chemical property, the RNC adopted a combination of $$^{51}$$V as the beam and $$^{248}$$Cm as the target, aiming to synthesize element Z$$=119$$ through the hot fusion reaction of $$^{51}$$V$$ +^{248}$$Cm. Here, the 3*n* and 4*n* reaction channels can be utilized leading to isotopes, namely, $$^{296}$$119 and $$^{295}$$119, respectively. Both reaction channels are expected to have excellent $$\alpha $$ decay chains emitting seven $$\alpha $$ particles consecutively (seven generations). The fact that the last five generations of them are known experimentally [9] can assist in identifying the Z = 119 event [10].

Table 1 lists the recent theoretical calculations of the evaporation residue (ER) cross-section $$\sigma _{\textrm{ER}}$$ of the $$^{248}$$Cm ($$^{51}$$V,$$xn)^{299-x}$$ 119 reaction for the $$x=$$3 and 4 channels. Although the theoretical prediction strongly depends on the assumed theoretical models, $$\sigma _{\textrm{ER}}$$ can be in the order of 10 fb or less. Therefore, intensive effort to increase the reaction yield is essential, such as the provision of an intense $$^{51}$$V beam, a $$^{248}$$Cm target resistant to high-heat load, or an efficient detection system including a focal plane detector (FPD).

**Table 1:** Theoretical calculations of the evaporation residue for $$^{248}$$Cm($$^{51}$$V,$$xn)^{299-x}$$119. The maximum cross-section of each channel is shown

$$^{248}$$Cm( $$^{51}$$V,$$xn)^{299-x}$$119	Cross section (fb)		
channel *x*	3*n*	4*n*	References
Ghahramany (2016)	20	100	[11]
Zhu (2016)	6	11	[12]
Adamian (2018)		12	[13]
Manjunatha (2019)	4		[14]
Siwek-Wilczynska (2019)	3	6	[15]
Aritomo (2020)	20 at E$^*$=20 MeV		[16]
Lv (2021)	9.8	1.3	[17]

The approximate bombarding energy required for a $$^{51}$$V beam to synthesis Z=119 via the $$^{51}$$V$$ +^{248}$$Cm reaction can be estimated through a simple Coulomb barrier height calculation assuming appropriate radii for $$^{51}$$V and $$^{248}$$Cm or by utilizing the systematic trend of the barrier height distribution measurements on $$^{248}$$Cm by Tanaka et al. [18, 19]. Both estimates indicate that the required beam energy of $$^{51}$$V is approximately 6 MeV/*u* ($$\sim \! 300$$ MeV) in the lab system.

Thus, the primary focus of the SHE project is the upgrade of the RILAC accelerator by replacing in part a superconducting RIKEN linear accelerator (SRILAC) to increase the final beam energy from 5.5 MeV/*u* to 6.5 MeV/*u* and building a new superconducting electron-cyclotron-resonance ion source (SC-ECRIS) operating at a higher RF frequency to increase the beam current.

GARIS, used for isolating and detecting nihonium events, was designed with a high-transmission efficiency for the recoiling evaporation residue produced in a cold fusion reaction. However, its transmission efficiency is significantly reduced for the evaporation residues produced in hot fusion reactions due to their lower recoil velocity compared to cold fusion reactions. Therefore, a new gas-filled recoil ion separator GARIS-II suitable for hot-fusion reaction products was designed and constructed [20]. It was installed next to GARIS in the experimental hall (see Fig. 1) in 2013, and its basic optical properties were examined using beams. The first trial experiment to synthesize superheavy-element oganesson was performed using RILAC and GARIS-II via the hot fusion reaction $^{50}$Ti$+^{248}$Cm$\rightarrow ^{298}$Og$^*$ in 2017. The SHE project aimed at synthesizing element 119 was approved during this period.

It was estimated that at least three years would be required for the construction of SRILAC. To avoid research interruption during the SRILAC construction period and gain insights on the hot-fusion reaction, GARIS-II was moved to experimental hall E6 in Nishina building (see Fig. 1) in 2018.

The existing accelerators, the injector linac (called RILAC2) and the RIKEN Ring Cyclotron (RRC), together with the vital SC-ECRIS rendered certain exploratory measurements possible using an intense $^{51}$V beam up to the end of 2019.

The upgrade project concluded with the newly constructed GARIS-III in the experimental hall in LINAC building where GARIS-II was previously located. GARIS-III is essentially a copy of GARIS-II.

In 2020, commissioning of the entire system (SC-ECRIS, SRILAC, and GARIS-III together with the newly built beamline) commenced. SC-ECRIS and SRILAC completely satisfied the planned specifications.

The upgraded accelerator system was designed to deliver a $^{51}$V beam with a current intensity of a few p$\mu$A at the target position of GARIS-III. However, the $^{248}$Cm target or FPD system may not be able to withstand such an intense beam. Study of the target backing material, in particular, for sustaining this intense beam for a reasonable period is urgently required. The new SHE search experiment commenced with trial-and-error under an international collaboration called the nSHE^1^ research group.

This contribution describes the SHE project at the RNC, which commenced in 2016, with particular emphasis on the accelerator facility upgrade (Sect. 2). This is followed by a short description of the transfer of GARIS-II to E6 (Sect. 3). Further, the construction and installation of GARIS-III (Sect. 4) and the commissioning of GARIS-II and GARIS-III (Sect. 5) are detailed. The $^{248}$Cm target preparation is then presented (Sect. 6). Finally, the recent research activities regarding the search for new element Z=119 are briefly described (Sect. 7), followed by the summary.

## Energy and intensity upgrade of RILAC

### Overview of the upgrade plan

**Fig. 2:**
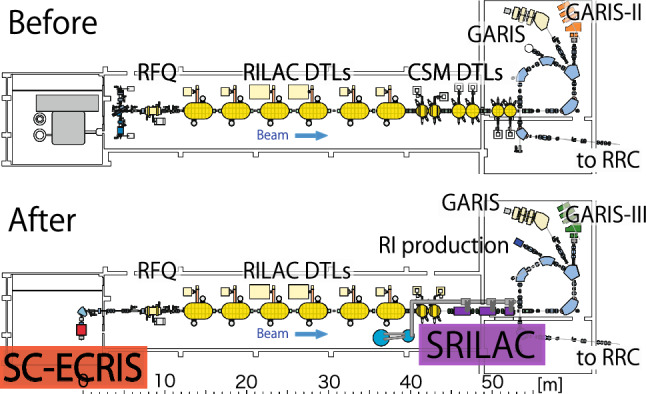
Overview of RILAC and SRILAC

The original RILAC [2] comprised six drift-tube-linac (DTL) tanks (see Fig. 2) that are frequency-tunable from 17–45 MHz and can accelerate heavy-ions to 2.9 MeV/*u* at 37.75 MHz as an injector for the RIBF accelerator complex [21].

Element Nh was synthesized by bombarding a $^{209}$Bi target with a $^{70}$Zn$^{14+}$ beam having an intensity of 0.5 p$\mu$A 2 and an energy of 352.6 MeV (5.04 MeV/*u*) [3]. The energy was increased from 2.9 MeV/*u* to 5.5 MeV/*u* by adding a booster linac called charge-state-multiplier (CSM) system [22] comprising six DTLs.

For the synthesis of a new SHE beyond oganesson, a heavy-ion beam with more than 6 MeV/*u* is required, necessitating energy upgrade. Therefore, a superconducting-linac (SC-linac) was proposed [23] for increasing the beam energy from 3.6 MeV/*u* to $\sim \!$ 6.5 MeV/*u*. Given the limited space in the existing RILAC building, SRILAC was installed by replacing four CSMs (see Fig. 2). The upgrade goals are listed in Table 2.

**Table 2:** Goal specifications for RILAC before and after upgrade

Upgrade	Before	After
Number of tanks	12DTLs	8DTLs,10SC–QWRs
Frequency (MHz)	37.75/75.5	36.5$^*$/73.0
Total Acc. *V* (MV)	25 (*A*/*q* = 5)	39 (*A*/*q* = 6)
Beam current (p$\mu$A)	0.5	>2.5

$^*$36.5 MHz is the fundamental frequency of the RF system of the RIBF accelerators

A new SC-ECRIS was constructed [24] to increase the beam current by at least five times than that used in the Nh synthesis experiment. The ion-source is a duplication of the RIKEN 28-GHz SC-ECRIS [25] developed for producing an intense uranium beam together with a new injector linac (RILAC2) [27] for the RIBF.

The construction of SRILAC and SC-ECRIS commenced in 2017.

### Energy upgrade (SRILAC)

#### Superconducting quarter-wave resonators for SRILAC

Superconducting RF technology is a mature technique that utilizes SC cavities composed of pure niobium, which are cooled to the liquid-helium temperature. Recently, large-scale SC-linacs for heavy-ions have been constructed at FRIB [28], SPIRAL2 [29], RAON [30], HIAF [31], HELIAC [32], and other laboratories.

At the RNC, the SC-linac design with 14 cryomodules (CMs) was also studied for the RIBF to gain more beam power up to 11 MeV/*u* for the uranium beam [33, 34].

**Fig. 3:**
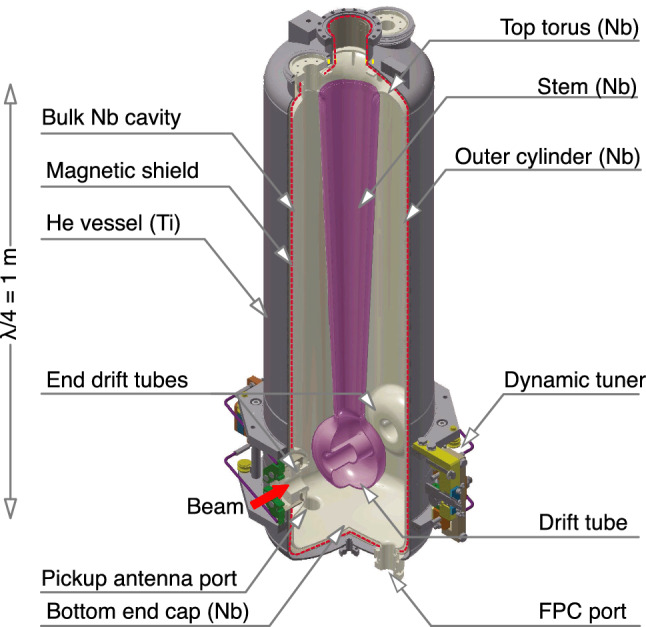
Schematic of the SC-QWR for SRILAC

The schematic of the SC-quarter-wavelength resonator (SC-QWR), based on this SRILAC design, is shown in Fig. 3.

**Fig. 4:**
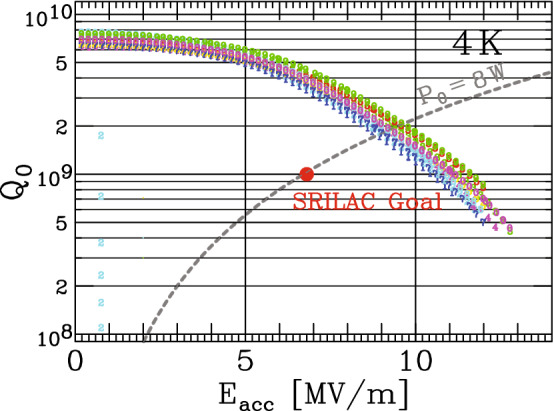
Results of the cavity performance validation tests. The dashed curve corresponds to the constant power dissipation of 8 W. All the ten bulk cavities exhibit similar behavior

SC-QWRs are made from highly purified Nb sheets. The bulk Nb cavity consists of an outer-cylinder, a stem, and top- and bottom- end caps welded through electron-beam-welding. After welding, the inner surfaces are treated by a standard processing method based on buffered chemical polishing and high-pressure rinsing with ultrapure water. Before installing a local magnetic shield (indicated by the red-dashed line in Fig. 3) and a helium vessel, validation testing was performed for all the SC-QWRs [35]. The cavity performance was validated by measuring the quality factor ($\hbox {Q}_0$) related to the power dissipation. It is defined as $\textrm{Q}_0 = \omega _0$ $U/P_0$, where *U* is the stored energy, $P_0$ is the power dissipated in the cavity walls, and $\omega _0$ is 2$\pi$ times the frequency. In addition, the narrowness of the resonance curve, which indicates the stored energy as a function of the RF frequency with a constant input RF power, characterizes the Q factor as $\Delta f/f_0 = \textrm{Q}$. For example, $\Delta f=0.073$ Hz for a resonant curve with $\hbox {Q}_0=1\times 10^9$ at a frequency of 73 MHz. In Fig. 4, the $\hbox {Q}_0$ values of the ten bulk cavities measured at 4.2 K are plotted as a function of $E_{\textrm{acc}}$. Thus, all the SC-QWRs completely satisfy the targeted $\hbox {Q}_0$ of $1\times 10^9$, which corresponds to $P_0$ = 8 W at an operational $E_{\textrm{acc}}$ of 6.8 MV/m.

The SC-QWR specifications for SRILAC are summarized in Table 3.

**Table 3:** SC-QWR specifications for SRILAC

Frequency at 4.5 K (MHz)	73.0 (c.w.)
$\beta _{\textrm{opt}}$	0.078
Max. acc. gradient $E_{\textrm{acc}}$ (MV/m)	6.8
${\textrm{R}}_{\textrm{sh}}/\textrm{Q}_{0}$ for $\beta _{\textrm{opt}}$ ($\mathrm{\Omega }$)	579
$G(\equiv \mathrm{Q_0}/R_{\textrm{surface}})$ ($\mathrm{\Omega }$)	22.4
Target $\mathrm{Q_0}$ at $E_{\textrm{acc}}=6.8$ MV/m	$1\times 10^9$
$P_0$ (W)	< 8
Frequency tuning range (kHz)	14

Among the various SC materials, extremely pure niobium is extensively used for SC cavities. Niobium has a relatively high critical temperature of $T_c$ = 9.25 K compared to the liquid-helium temperature and a critical magnetic field of $B_c$ = 200 mT. Moreover, the physical and mechanical properties of niobium resist the helium pressure and the deformation produced by the frequency tuning system, which squeezes the cavity resulting in elastic deformation. The dynamic frequency tuner, which reduces the gap between the drift tubes, achieves a tuning range of 14 kHz without giving plastic deformation. Note that one of the characteristics of the SC-QWR is tilted-angle-facing is adopted to its acceleration gaps to compensate for the steering effect caused by the RF magnetic field [36]. Each SC-QWR is equipped with a fundamental power coupler (FPC). The FPC is a single-window coaxial-type, which comprises a stainless-steel outer conductor to minimize the thermal load to the SC-QWR, a copper antenna to reduce the RF power dissipation, and a ceramic window located in the room-temperature part.

#### Cryomodule (CM)

**Fig. 5:**
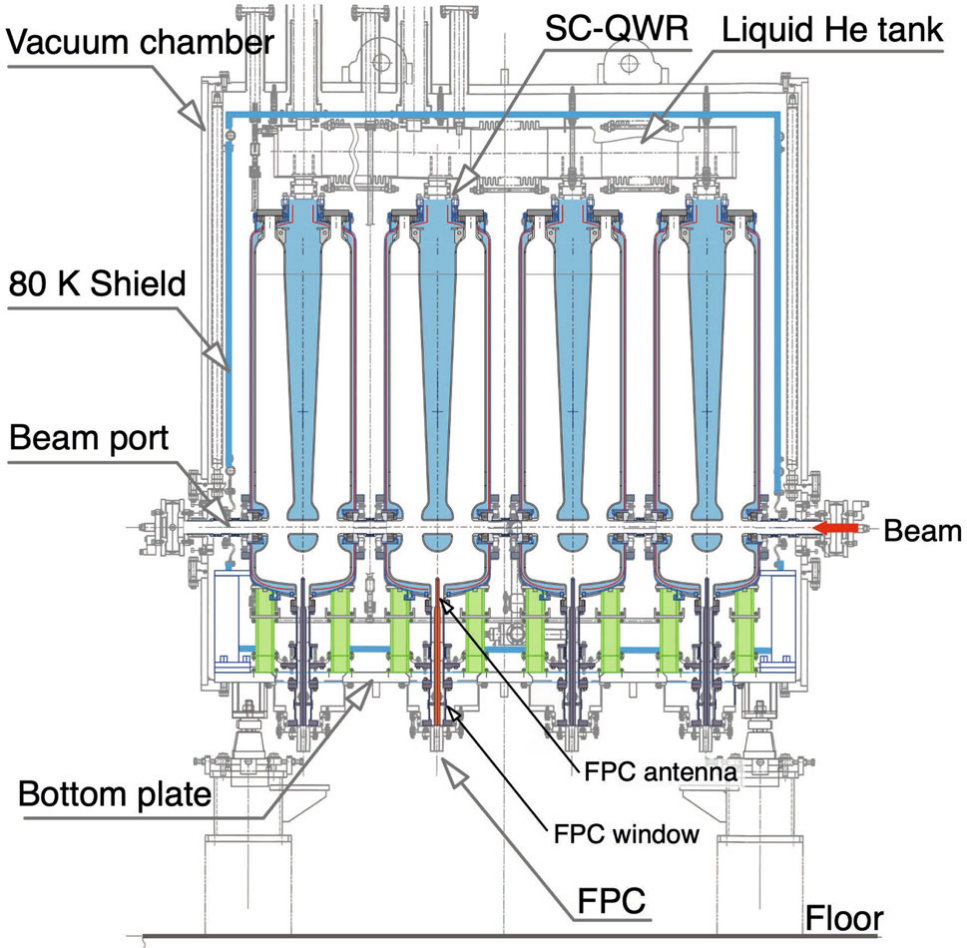
Side-view of the cryomodule

SRILAC comprises three CMs (CM1, CM2, and CM3) and a medium-energy beam-transport-line (MEBT), which connects the CMs, RILAC DTLs, and the high-energy beam transport line. Both CM1 and CM2 contain four SC-QWRs each. The schematic of CM1 and CM2 is shown in Fig. 5.

The design of CM3 is the same as that of CM1 and CM2 but it contains only two SC-QWRs. Instead of the traditional top-loading type, bottom-up-type CMs are used. The 4K cold mass, which comprises a cavity, a Mu-metal local magnetic shield, a helium vessel, FPC, and a dynamic tuner (Fig. 3), is supported by pillars composed of G10 (a high-pressure fiberglass lamination) from the bottom-base plate and housed in a vacuum chamber. One of the advantages of the bottom-up structure is that it is easier to align the cavities considering the shrinkage of the cavity supports cooled to 4K, compared to the top-loading type. Thermal shields cooled with liquid nitrogen are installed to minimize the heat-load to the 4 K part from the room-temperature part. Each cavity is connected to the adjacent cavities through beam bellows for separating the cavity vacuum from the insulation vacuum. The CMs were assembled in an ISO class 1 cleanroom facility to prevent the entry of dust into the cavity.

#### RF control

The FPC is designed with tunable coupling to achieve an external quality-factor ($\hbox {Q}_{\textrm{ext}}$) ranging from 1 $\times 10^6$–4.5 $\times 10^6$ by changing the insertion distance of its antenna. $\hbox {Q}_{\textrm{ext}}$, defined as the ratio of the initial energy stored in the cavity to the energy lost in one radian of the oscillation cycle, was selected as $1 \times 10^6$ with an over-couple condition because it is difficult to maintain the resonant frequency of each SC-QWR within ± 0.073 Hz. In this case, an output power of 7.5 kW is required for the RF amplifiers to realize an operational bandwidth of ± 60 Hz, while the dissipated power on the cavity wall is expected to be less than 8 W. Nevertheless, the antenna is not geometrically shorted at the cavity end; the input impedance becomes a short-circuit and the RF current at the cavity antenna becomes twice the forward RF current.

Cavity tuning by adjusting the resonant frequency to the operating frequency is accomplished by squeezing the cavity mechanically along the beam axis, as previously mentioned. For a frequency tuning of 14 kHz, $\Delta L$ = –0.37 mm with a force of 7.5 kN is required. Moreover, the tuner finely compensates for the frequency variation due to Lorentz force detuning, which is a resonant frequency change due to the mechanical deformation of the cavity by an electromagnetic force, and helium pressure deviation $\Delta f/ \Delta P_{\textrm{He}}$= –2.0 Hz/hPa within ± 60 Hz.

The signal from the pickup antenna follows the amplitude and phase of the excited RF voltage. The ratio of the amplitude of the pickup signal to the excited RF voltage was determined through the cavity validation test. A low-level RF circuit compensates for the amplitude and phase errors caused by resonant frequency change due to the mechanical vibration of the cavity (microphonics), pressure change of the supplied helium, and Lorentz force detuning.

#### Medium energy beam transport (MEBT)

The function of MEBT is to maintain the vacuum, transversely focus the beam, and perform beam diagnostics.

For beam transport, room-temperature quadrupole magnets, which include horizontal and vertical steering functions, are used.

Instead of traditional beam diagnostic devices, such as wire scanners and Faraday cups, the beam-energy-position monitor, named BEPM, is employed. The BEPM is intended to simultaneously measure the timing, position, energy, longitudinal profile, and amplitude of the beam. Eight BEPMs are installed to measure the beam properties before and after acceleration by each CM. The BEPM for SRILAC was developed along with the Beam Diagnostics Group of J-PARC [37].

As beam measurement is nondestructive, ideally, there is neither outgassing nor spattering to produce dust, which degrade the field emission on the superconducting cavity surface.

One of the most critical issues in the design of the beam transport line is the prevention of SC-cavity contamination by the dust transported from the room-temperature section through gas flow due to the vacuum pressure gradient. While the vacuum-pressure level of the SC part drops as low as 1 $\times 10^{-8}$ Pa, the vacuum pressure in RILAC, designed and built more than 40 years ago (first beam in 1981), is 1 $\times 10^{-5}$ Pa–1 $\times 10^{-6}$ Pa. To connect different vacuum-level parts and prevent gas flow into the high-vacuum section, a nonevaporable-getter-based differential pumping system was developed [38]. This differential pumping system sandwiched CMs (not shown) reduces the pressure from the vacuum of the existing beamline of RILAC to the ultrahigh vacuum in SRILAC.

### Intensity upgrade (RIKEN 28-GHz SC-ECRIS)

**Fig. 6:**
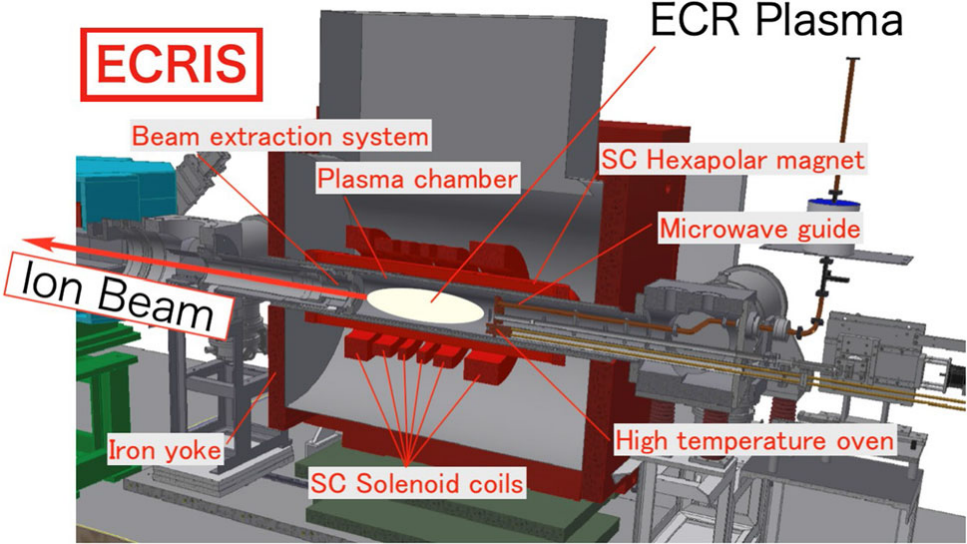
Cut-view of the RIKEN 28-GHz SC-ECRIS (Courtesy Y. Higurashi)

The new SC-ECRIS is structured to generate a powerful magnetic mirror for confining the extremely hot electrons heated by high-power microwaves. As shown in the schematic (Fig. 6), the RIKEN 28-GHz SC-ECRIS comprises six SC solenoidal coils and a hexapolar SC magnet to achieve a sufficiently large mirror ratio of $B_{\textrm{max}} / B_{\textrm{min}} \sim \! 4$, where $B_{\textrm{max}}$ and $B_{\textrm{min}}$ are the maximum and minimum magnetic fields, respectively, with $B_{\textrm{max}}$ = 4 T.

In addition, using the six SC solenoidal coils, it is possible to generate various mirror field distributions along the ion-source axis, for example, either “classical $B_{\textrm{min}}$” or the so-called “flat $B_{\textrm{min}}$” [39]. This magnetic configuration enables the confinement of the high-density ECR plasma within a large-volume plasma chamber of 10 L. Because the large volume of the ECR plasma increases the number of collisions between the atoms or ions and the energetic electrons in the plasma, the production of multiply charged ions increases. From our experience, the two-frequency injection method using two RF power sources with frequencies of 18 GHz and 28 GHz improves the stability of the extracted beams [40].

For long-term operation at the RIBF, the ion-source operation is optimized to minimize the X-ray heat load, which destroys the insulation materials in the cryostat chamber.

To provide metal ions such as uranium or vanadium for a period of one month, an ion-source structure with a pair of high-temperature ovens was developed and installed [41]. An intense $^{51}$V$^{13+}$ beam is being produced, which exceeds the targeted beam current for the experiment.

### Beam commissioning

After the hardware was commissioned, in January 2020, the beam acceleration test was conducted for the first time. The specifications of SRILAC are listed in Table 4.

**Table 4:** SRILAC specifications

Frequency (MHz)	73.0 (c.w.)
$E_{\textrm{inj}}$ (MeV/*u*)	3.6
$E_{\textrm{ext}}$ (MeV/*u*)	6.5 for *A*/*q* = 6
Max. gap voltage (MV/cavity)	2.4
Synchronous phase ($^\circ$)	–25
Max. acc. gradient (MV/m)	6.8
Liquid He temperature (K)	4.5
Beam current ($\mu$A)	$\le$100
$\hbox {Q}_{\textrm{ext}}$	1–4.5 $\times 10^6$
Amplifier output (kW)	7.5

**Fig. 7:**
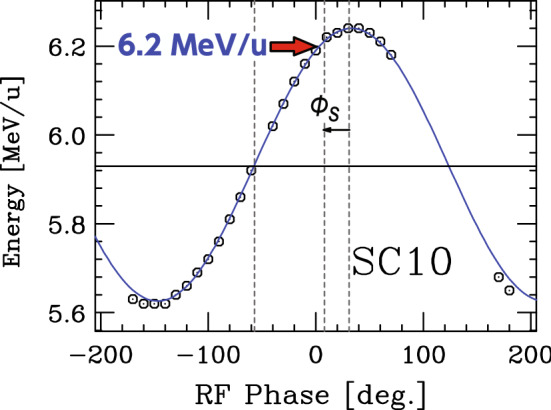
Phase scan plot where $\hbox {E}_{\textrm{out}}$ is plotted as a function of the RF-field phase for SC10. The targeted energy of 6.2 MeV/*u* is indicated by a red arrow

An $^{40}$Ar$^{13+}$ beam with an intensity of approximately 23 enA (duty 3%, chopper frequency 1 kHz) was accelerated to 6.2 MeV/*u* with a gap voltage of 1.13 MV/cavity. For the acceleration test, only nine SC-cavities were used because there was a vacuum leak with the ceramic window of the FPC of cavity No. 5 (SC05) then. For SC-linac tuning, the SC-QWRs were energized one-by-one, and the beam energy was measured, with the systematic variation of the RF-field phase (see Fig. 7).

The beam energy was precisely measured through the time-of-flight (ToF) method using a pair of BEPMs with low-beam current. The RF phase of each cavity must be synchronized with the timing of the beam bunches. Then, the energy gain of the beams passing through the cavity can be denoted as $\Delta E = V_0 \cos (\phi _{\textrm{s}})$. Here, $V_0$ and $\phi _{\textrm{s}}$ are the acceleration voltage and synchronous phase, respectively. For SRILAC, $\phi _{\textrm{s}} = -25^\circ$ was obtained by shifting the RF-field phase from the zero acceleration/bunching phase by 65$^\circ$ toward the top of the cosine curve. The beam positions, which were monitored by the BEPMs, were maintained at almost the center of the beam aperture during the phase scan through geometrical correction by the steering effect of the QWR cavity. Finally, the accelerated beam energy reached 6.2 MeV/*u* at 9 pm on January 28th, 2020. After careful tuning, the transmission efficiency through the SRILAC section from the entrance to exit reached $\sim \!$100% with a beam current of 6.11 e$\mu$A.

The vertical and horizontal beam positions monitored by the BEPMs were finely centered; the beam loss, which mainly occurred in MEBT, was minimized, keeping the deterioration of the vacuum pressure below 1$\times 10^{-7}$ Pa due to beam loss.

After beam commissioning and the mitigation of various problems, user beam service commenced. A $^{51}$V$^{13+}$ beam with energy ranging from 4.2–6.3 MeV/*u* was accelerated and delivered. Stable beams with the required energy and intensity were successfully provided after careful tuning, and a beam power of more than 1 kW was achieved.

From the accelerating structure point-of-view, one of the advantages of the double-gap cavity, adopted in the SC-QWR for SRILAC, is the high-flexibility in accelerating the beam energy compared to multicell drift tube linacs. Utilizing the independently phased array of the QWR, the accelerating beam energy can be set within a wide range and finely tuned by selecting the number of cavities and gap voltages. For example, the acceleration of light-ions, such as alpha particles, is possible at 7.25 MeV/*u*, which is the optimal energy for producing $^{211}$At through the $^{209}$Bi($\alpha , 2n)$ $^{211}$At reaction for targeted radionuclide therapy. A dedicated beamline for $^{211}$At production is under construction.

Thus far, a $^{51}$V beam current of 3.5 p$\mu$A has been achieved on target. The beam losses in the SRILAC section caused by various errors, such as acceleration voltage and phase errors or magnet power supplies, are well under control, and do not exceed 1 W/m. Continuous efforts are being made to increase the beam current by improving the stability of the acceleration RF field, stability and reliability of the magnet power supplies, and the transmission efficiency of the low-energy-beam transport section.

## GARIS-II transfer to E6

GARIS-II in the RILAC experimental hall in the LINAC building, which had been operational since 2013, was transferred in 2018 to the E6 experimental hall in Nishina building together with the gas-cooled rotating target system, differential pumping system, FPD system, and analog electronics.

At the new location in E6, the available area for GARIS-II was limited by the floor and wall structures. Therefore, the GARIS-II magnets were reversed to change the ion deflection angle from left to right for fitting into the available floor space. Dedicated beam diagnostics for GARIS-II were newly installed.

One of the booster linac CSMs used in RILAC, which was decommissioned for adopting to SRILAC, was transported in the beam transport line of GARIS-II. It was used for modifying a small quantity of beam energy, such as ± 3%. In addition, a ToF system was installed to measure the beam velocity. The entire relocation process required half a year for completion.

In parallel with the relocation, the RRC was modified to accelerate $^{51}$V ions to an energy of approximately 6 MeV/*u* for the SHE experiments. However, in the standard RRC operation for RIBF experiments, ions are accelerated to an energy of 10.7 MeV/*u* by the combined operation of RILAC2 and RRC at an RF frequency of 18.25 MHz and harmonic number (H) nine. To match a specific energy of $\sim \!$ 6 MeV/*u* for the SHE experiments, H = 12 was selected. One of the operational issues for H = 12 is that the acceleration voltage of the RF cavity (frequency-tunable double gap type) is as low as 60 kV/gap at an RF frequency of 18.25 MHz. This acceleration voltage is insufficient for handling high-power beams with the space charge effect. To solve this problem, the two double-gap resonators [42] were remodeled, and an acceleration voltage of 120 kV/gap was successfully achieved.

By 2018, SHE experiments could commence using the accelerator complex, 28-GHz ECRIS, RILAC2 and RRC, and the transferred GARIS-II system.

## Construction and installation of GARIS-III

**Fig. 8:**
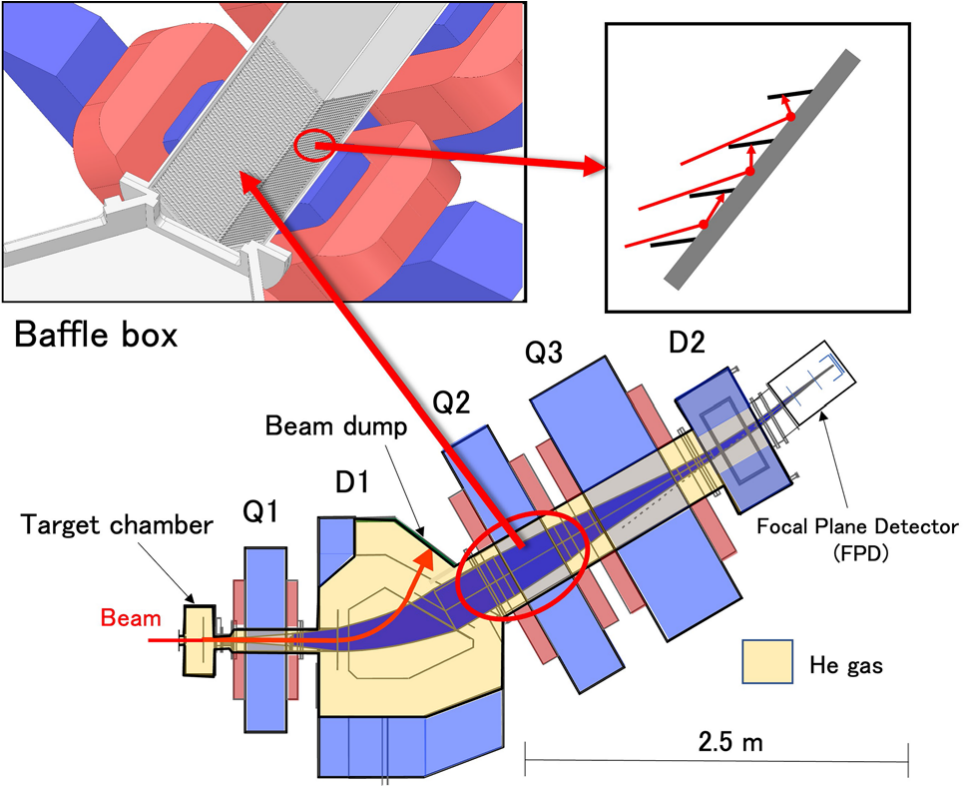
Plan view of GARIS-III from Ref. [44]. Inset on the top-left is the 3D cross-sectional drawing of the baffle box within the vacuum chamber at the Q2 position. The baffle fin, magnet coil, and iron yoke are indicated in dark gray, reddish-brown, and blue, respectively. Inset on the top-right is the cross-section of the baffle with certain background particle trajectories as an example

By the time SRILAC was completed, GARIS-III was constructed and installed in the RILAC experimental hall to conclude the main part of the SHE project. Note that GARIS-III is a duplication of GARIS-II and is placed in the previous location of GARIS-II next to GARIS. The magnet configuration for both GARIS-II and GARIS-III includes two dipole magnets (D1 and D2) and three quadrupole magnets (Q1, Q2, and Q3) as shown in Fig. 8.

However, a minor modification was made based on the operational experience of GARIS-II for reducing the background events at the focal plane. These background events are partially caused by rescattering from the inside surface of the D1 vacuum chamber. A rectangular (280 mm$\times$280 mm) baffle box [43] with a length of 650 mm was placed in the vacuum chamber at the Q2-magnet of GARIS-III, as depicted in the inset on the top-left of Fig. 8. All the four walls (left-up, left-down, right-up, and right-down) within the baffle box were covered with fins separated by 12 mm. These 7-mm wide fins are composed of stainless steel and inclined at 45$^{\circ }$ to the wall. Part of the cross-sectional view of the fins in the baffle box is shown in top-right inset in Fig. 8.

Subsequently, the effect of the baffle box in GARIS-III was investigated by measuring the background count rates of GARIS-III and GARIS-II (without the baffle box) at each focal plane. It was found that the background count rate of GARIS-III was reduced by $\sim$50% for a particle energy $> 1$ MeV compared to that of GARIS-II.

The design, construction, and performance of GARIS-II are reported in Ref. [44]. The ion optical properties of GARIS-II were studied through two $$^{40}$$Ar-induced fusion reactions, $$^{169}$$Tm($$^{40}$$Ar,$$4n)^{205}$$Fr and $$^{208}$$Pb($$^{40}$$Ar,$$3n)^{245}$$Fm [45]. Furthermore, GARIS-II was used for investigating the decay properties of $$^{283}$$Cn [46]. Accordingly, the commissioning work for GARIS-III was curtailed; therefore, only basic properties such as the ion-optics and solid angle were confirmed as shown in Sect. 5.

Only the new aspects associated with the installation of GARIS-III are described below.

### Beamline to GARIS-III and the target chamber

**Fig. 9:**
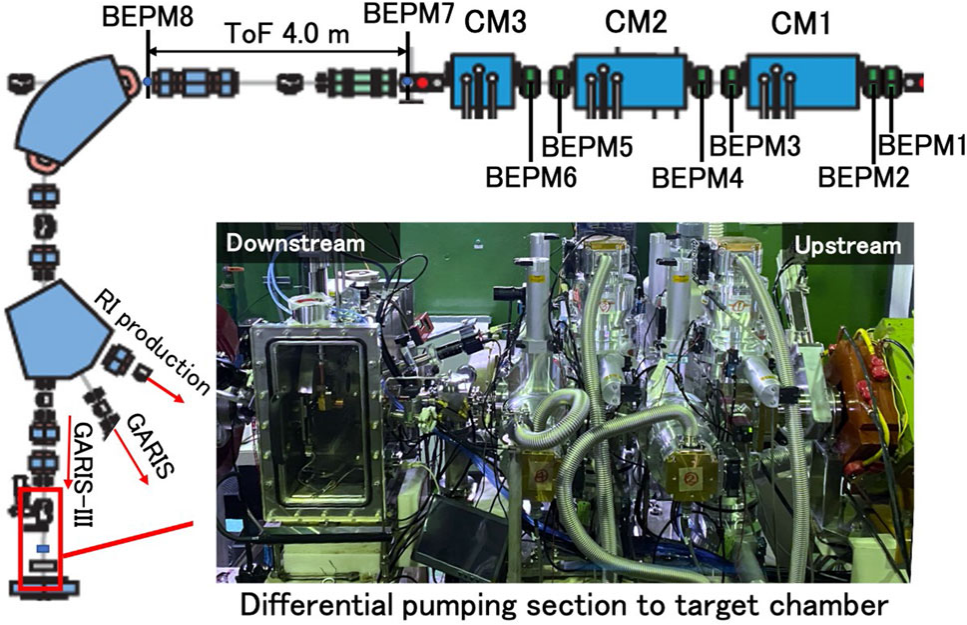
Beamline from SRILAC to GARIS-III. Inset is the photograph of the red-square-box area depicting a part of the beamline from the differential pumping section to the Q1 magnet of GARIS-III

Figure 9 shows the layout of SRILAC including CM1, CM2, CM3, eight BEPMs and the beamline to GARIS-III. The beam from SRILAC can be switched to GARIS-III, GARIS, and the RI production apparatus depending on the application.

**Fig. 10:**
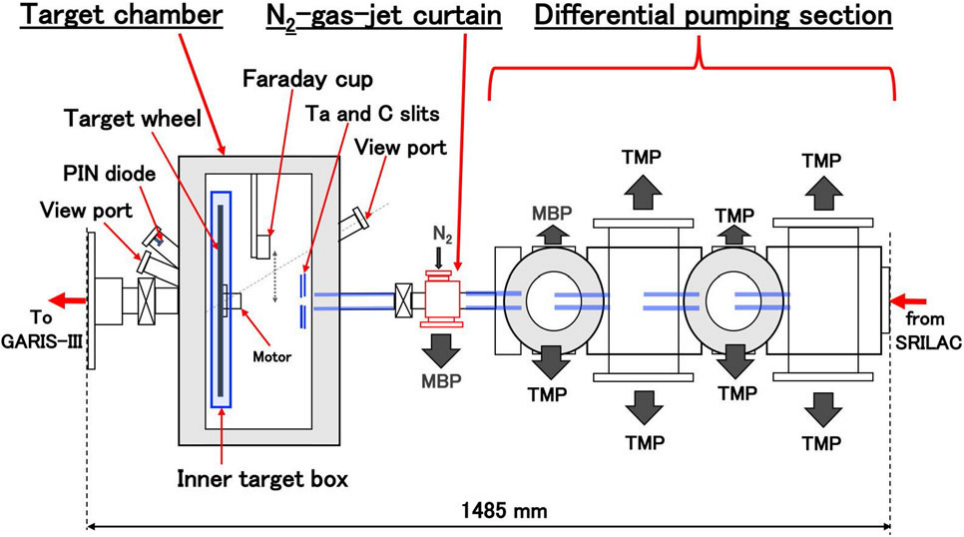
Layout of the differential pumping section, $\hbox {N}_2$-gas-jet curtain, and target chamber

The inset photograph in Fig. 9 shows the differential pumping section (DPS), $\hbox {N}_2$-gas-jet curtain [47], target chamber, and Q1 magnet of GARIS-III from left- to-right. The DPS and target chamber designs are the same as those in GARIS-II. For further details, see Ref. [44, 45]. The layout corresponding to the inset photograph in Fig. 9 is shown in Fig. 10.

#### ToF measurement to derive the beam velocity

Between the exit of SRILAC (CM3) and the 90$^{\circ }$ bending magnet, there is a 4-m straight section as shown in the top of Fig. 9. This section serves as the flight distance of ions to measure the beam velocity through the ToF method utilizing the BEPMs located at the entrance (BEPM7) and exit (BEPM8) of this straight section as indicated in Fig. 9. The accuracy of the measured beam velocity was cross-checked with the value by the momentum analysis with the 90$^{\circ }$ bending magnet. Even though the beam intensity is as low as 20 enA, the beam position and energy can be simultaneously measured to accuracies of $\pm 0.1$ mm and precision of $\sim 10^{-3}$, respectively [37]. This energy precision, considering the uncertainty of beam energy spread ($\Delta E/E\sim \pm 0.2\%$ in rms), is sufficient for our purpose.

#### Differential pumping section (DPS)

In the DPS, seven turbomolecular pumps (TMP) with an evacuation speed of 350 l/s and a mechanical booster pump (MBP) with an exhaust rate of 280 l/s are employed. In addition, an MBP with an exhaust rate of 280 l/s is attached to the $\hbox {N}_2$-gas-curtain apparatus. Among the five 25-mm diameter orifices indicated in blue in Fig. 10, four are 100 mm long, whereas the one connected to the target chamber has a length of 300 mm.

#### $\hbox {N}_2$-gas-jet curtain

Because the differential pumping system has a poor pumping speed for He gas, a device called ‘$\hbox {N}_2$-gas-jet curtain’ was newly included between the differential pumping section and target chamber (filled with 30–100 Pa of He-gas). This reduces the load to the differential pumping section for achieving better vacuum and preventing He-gas flow to the superconductive cavity. The $\hbox {N}_2$-gas-jet curtain position is indicated in Fig. 10.

The $\hbox {N}_2$-gas-jet curtain is highly effective and reduces the pressure at the exit of the CM3 from $\sim \!10^{-7}$ Pa to $\sim \!10^{-8}$ Pa.

Charge-state variation of the evaporation residues was not observed in GARIS-III after the installation of the $\hbox {N}_2$-gas-jet curtain. Moreover, an increase in the background was not observed in the FPD.

#### Target chamber

For the transfer of GARIS-II mentioned in Sec. 3, a new semiclosed inner-target box was fabricated to cope with the sizeable rotating target wheels with diameters of 10, 20, and 30 cm. The inner-target box, shown in Fig. 10, contains a motor system for rotation and a Cm target wheel, which can be adjusted up to 2000 rpm. This box can be mounted in the target chamber by inserting it from the side. In addition, it is expected to confine the precious Cm material within the inner-target box and prevent scattering in all the directions of the large-volume scattering chamber if the targets are broken by accident, to minimize loss.

A Faraday cup can be inserted at the target position to measure the beam intensity when necessary. The beam spot size is confirmed by observing the luminescence of the alumina fluorescent plate ($\hbox {Al}_2$–$\hbox {O}_3$) inserted in the target position, using a CCD camera, through the viewing port attached to the chamber.

During the experiment, the beam intensity was continuously monitored by measuring the elastically scattered projectiles by the target, with an Si positive-intrinsic-negative (PIN) photodiode (Hamamatsu S1223) mounted at 45$^{\circ }$ with respect to the beam axis as shown in Fig. 10.

At the entrance of the target chamber, there is a Ta slit with an aperture sized 11 mm$^{\textrm{H}}\times$9 mm$^{\textrm{V}}$. The slit is split into four plates (up, down, left, and right) enabling independent reading of the beam current. Immediately behind this Ta slit, a carbon slit sized 10 mm$^{\textrm{H}}\times$8 mm$^{\textrm{V}}$ is also set. These Ta and carbon slits contribute to beam-spot adjustment and maintenance under high-intensity beam current. Furthermore, they prevent the beams from hitting materials other than the target.

### FPD system

**Fig. 11:**
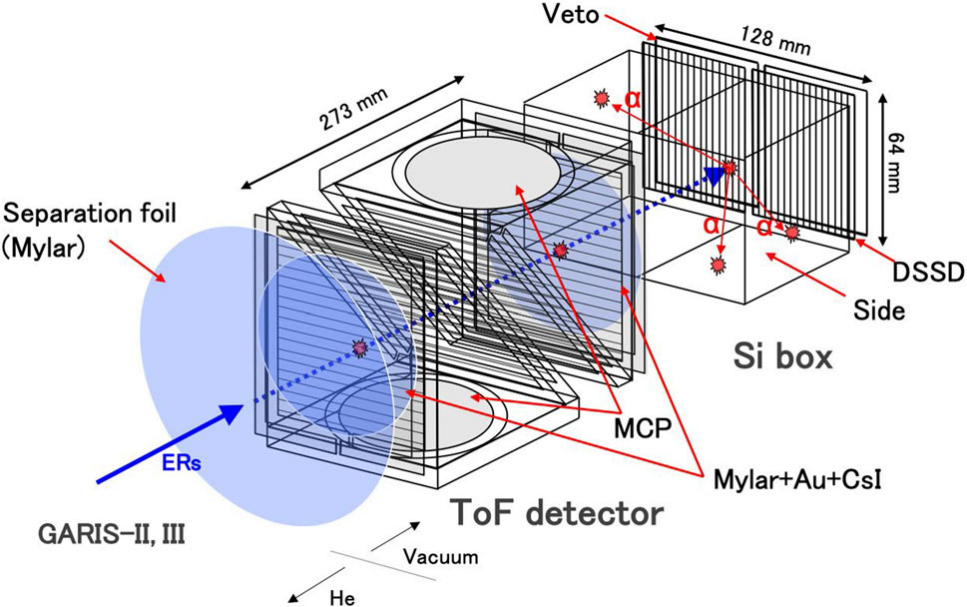
FPD system of GARIS-III

The FPD system depicted in Fig. 11 comprises a ToF detector system and an Si detector box.

The three blue circles shown in the figure are 0.5 $\mu$m-thick Mylar films. The large blue circle is an insulation window that separates the FPD in a vacuum from the gas-filled chamber of GARIS-III. The small blue circle is the entrance (exit) foil of the ToF detector, with a diameter of 140 mm. The entrance (exit) foil is coated with gold (19.3 $\mu$g/cm$^2$) on one side to serve as an electrode and cesium iodide (CsI, 20 $\mu$g/cm$^2$) to increase the secondary electron emission.

#### ToF detector at the focal plane

A newly developed ToF detector was installed and used in the FPD of GARIS-II located in the RILAC experimental hall, as shown in Fig. 1 of Ref. [45] before relocation to the E6 experimental hall. When GARIS-II was relocated, the ToF detector system was reinforced by adding another ToF detector to form a tandem ToF detector system, as depicted in Fig. 11. The two ToF detectors are arranged in a back-to-back configuration, and the entrance and exit foils are separated by 273 mm.

The aperture of the ToF detector used in GARIS-II was increased compared to that used in GARIS, from 57 cm$^2$ to 154 cm$^2$, to cope with the spread of the reaction residues due to the hot fusion reaction [48]. The performance of this newly developed large ToF detector was examined using the $\alpha$ particles from $^{241}$Am decay, having an energy of 5.486 MeV equivalent to the typical light-charged particle energy. The time resolution was found to be 0.59 ± 0.02 ns (FWHM) irrespective of the position. Moreover, the detection efficiency was investigated and found to range from approximately 96–99% when the applied high-voltage was optimized [48].

#### Si detector box

The Si detector box contains two double-sided silicon strip detectors (DSSDs) for implantation surrounded by a tunnel detector, and a Veto detector located behind the DSSD. Each DSSD with a thickness of 500 $\mu$m has a sensitive area of $64\times 64$ mm$^2$ divided by 64 X-strips and 32 Y-strips. All the DSSDs are coupled with custom-made fast charge-sensitive preamplifiers (CR-110 and CR-111 chips, CREMAT Inc.) [49]. The tunnel detector comprises six 300 $\mu$m-thick Si detectors (one on the right and left, and two at the top and bottom) with a sensitive area of $58\times 58$ mm$^2$. The Veto detector contains two 300 $\mu$m-thick Si detectors sized 58 mm$\times$58 mm.

**Fig. 12:**
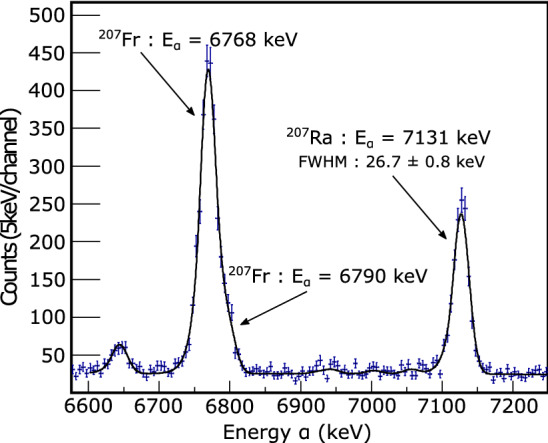
Energy resolution of the DSSD. See text for details

Two DSSDs arranged side-by-side cover the necessary sizes of the focal plane image (approximately 70 mm$\times$ 140 mm).

The energy resolution of each strip detector was determined by measuring the $\alpha$ decay spectrum induced by the $^{51}\textrm{V}+^{159}$Tb $\rightarrow ^{206}$Fr/$^{208}$Ra reaction. Figure 12 displays the observed $\alpha$ energy spectrum. The peak at 7.131 (6.915) MeV corresponds to the $\alpha$ decay of $^{207}$Ra ($^{205}$Fr). A tailed-Gaussian function form of Ref. [50] was applied for fitting the peak shape and the width was deduced. The typical energy resolution was approximately 27 keV (FWHM) at nearly 7 MeV but scattered within ± 5 keV depending on the strip [51].

It is to be noted that the time resolution of $\le$1 ns achieved by the tandem ToF detector system improved the reliability of distinguishing the evaporation residue implanted in the DSSD from the background events. The Veto detector also played an essential role in reducing background events. Note that the time resolution between a single ToF detector and DSSD used in the previous FPD system was approximately 5 ns.

#### DAQ system

In addition to the analog data-taking system, we introduced a digital electronics system [51] connected to the DSSD based on the Pixie-16 module (XIA LLC) [52] in collaboration with the University of Tennessee Knoxville (UTK) and Oak Ridge National Laboratory (ORNL). The pileup signal in the analog electronic system can be decomposed into two separate pulses.

**Fig. 13:**
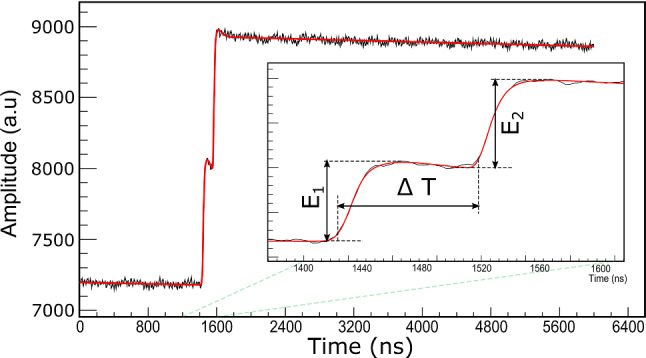
Decomposition of the pile-up pulse. See text for details

A typical double $\alpha$ pile-up pulse signal observed in the $^{51}\textrm{V}+^{248}$Cm run is shown in Fig. 13. Inset is the expansion of the region-of-interest. As shown in the inset, the double-pulse shape can be disentangled into two separate pulses enabling the extraction of the two pulse heights $\hbox {E}_1$ and $\hbox {E}_2$ separately. Such separation is possible down to a pileup signal overlapping each other in $\Delta$T$=$100 ns. This is a remarkable improvement because the analog system can only reach 5 $\mu$s [51]. Thus, the dead time loss is significantly reduced.

## Commissioning of GARIS-II and GARIS-III

At the beginning of commissioning, the transmission rate of the evaporation residues through GARIS-II(-III) was measured using the $$^{208}$$Pb($$^{40}$$Ar,$$3n ) ^{245}$$Fm reaction at 197 MeV whose cross-section is accurately known [53]. The reaction was identified based on the characteristic $$\alpha $$ and fission decay patterns. The obtained transmission was $$63\pm 9$$% for both GARIS-II [45] and GARIS-III [54].

Further, the excitation functions of the $$^{208}$$Pb$$(^{40}$$Ar,*xn*) $$ ^{248-x}$$Fm reactions for the $$x=2-4$$ evaporation channels were measured with GARIS-III [54]. The reaction channels were established using the respective characteristic $$\alpha $$ and fission decay patterns. The cross-sections were deduced from the transmission efficiency of 63%, $$\alpha $$ detection efficiency of 50% of the DSSD, and target thickness of 400 $$\mu $$g/cm$$^2$$.

**Fig. 14:**
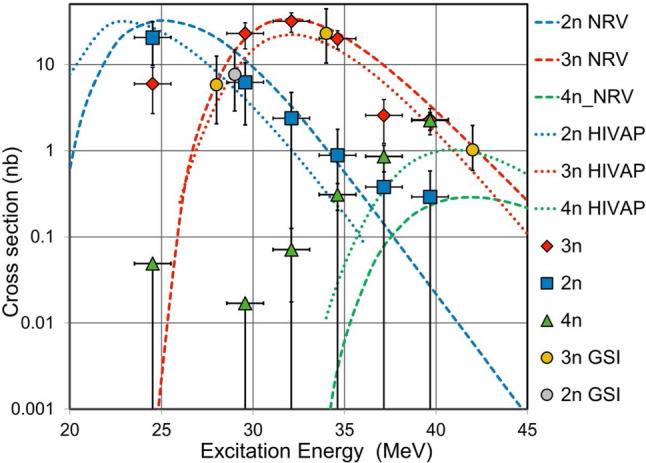
Excitation functions of the $$^{208}$$Pb$$(^{40}$$Ar,$$xn ) ^{248-x}$$Fm reactions. The results are from Ref. [54]. The reference data for the 2*n*- and 3*n*-channels are the GSI results [55]. The calculations are based on NRV and HIVAP. See text for details

The results of the excitation functions for the 2*n* to 4*n* evaporation channels are plotted in Fig. 14 along with those measured at GSI [55]. As both results are highly consistent, it can be concluded that the fundamental properties of GARIS-III are confirmed [54].

The obtained results for the $x=2-4$ channels were compared with the theoretical predictions by the nuclear reactions video (NRV) code [56] and the statistical fusion evaporation code, HIVAP [57]. The standard built-in parameters were used. It is interesting to note that that the above-mentioned calculations reproduce the experimental results well.

After the completion of GARIS-II, a commissioning experiment to synthesize element 118, oganesson, through hot fusion reaction $^{50}$Ti$+^{248}$Cm$\rightarrow ^{294,295}$Og was attempted before transferring GARIS-II from the RILAC experimental hall to the E6 experimental hall in Nishina building [58]. A $^{50}$Ti beam was generated using the 18-GHz ECRIS [59] through the MIVOC method arranged by the IPHC group in Strasbourg [60]. The result will be reported elsewhere [61].

## Target preparation

**Fig. 15:**
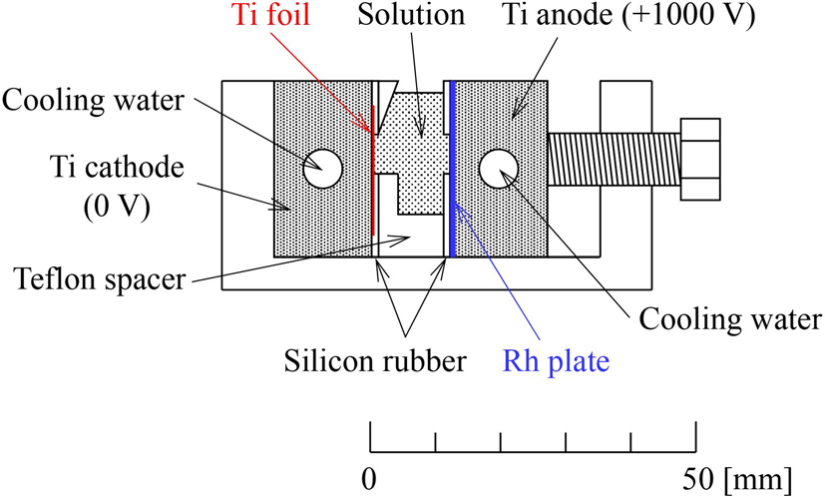
Schematic of the cell used for electrodeposition

Curium in an oxide form ($\hbox {Cm}_{2}\hbox {O}_{3}$) can endure high-intensity heavy-ion bombardment in prolonged beam irradiation. Large and uniform $^{248}\hbox {Cm}_{2}\hbox {O}_{3}$ targets for application in various GARIS experiments were successfully fabricated through a molecular plating method. Figure 15 shows the schematic of the cell used for electrodeposition at RIKEN [62].

**Fig. 16:**
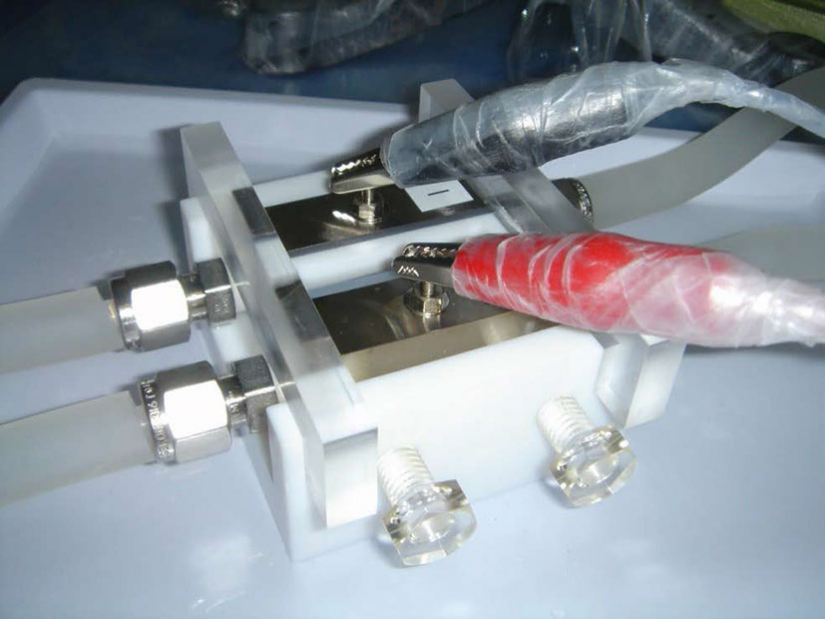
Photograph of the cell used for electrodeposition

A photograph of this cell is displayed in Fig. 16. A 2 $\mu$m-thick Ti backing foil placed on a water-cooled Ti block is employed as the cathode, whereas a 0.1 mm-thick Rh plate on another Ti block is used as the anode. A 10-mm-thick Teflon spacer perforated in an arc-shape is sandwiched between the Ti-block electrodes sealed with 1-mm-thick silicon rubber pieces perforated in the same arc shape. The active target area is 2.04 cm$^{2}$. The typical procedure for electrodeposition is given below, quoting an actual case.

The isotopic composition of Cm was $^{ 248}$Cm: 96.636%, $^{247}$Cm: 0.040%, $^{246}$Cm: 3.170%, $^{245}$Cm: 0.130%, and $^{244}$Cm: 0.024%. Twenty microliters of 0.2 M $\hbox {HNO}_3$ containing 610 $\mu$g of $^{248}$Cm were mixed with 5.5 mL of 2-propanol, and the electrodeposition cell was filled with the mixture. Electrodeposition of $^{248}$Cm in a nitrate form was performed by applying a voltage of 1000 V with an increase in the current density from 9.8 to 11.8 mA/cm$^{2}$ for 10 min. During electrodeposition, the Ti blocks were continuously water-cooled at 10$^{\circ }$C. After electrodeposition, the target was dried using an infrared lamp. The target thickness of $^{248}$Cm averaged for the six fabricated targets was determined to be 320 ± 20 $\mu$g/cm$^{2}$ through $\gamma$-ray spectrometry on $^{245}$Cm, referring to the isotopic composition of Cm. The average deposition yield was 100$^{+0}_{-3}$%. Finally, the Cm nitrate target was converted to oxide by heating through heavy-ion beam irradiation.

**Fig. 17:**
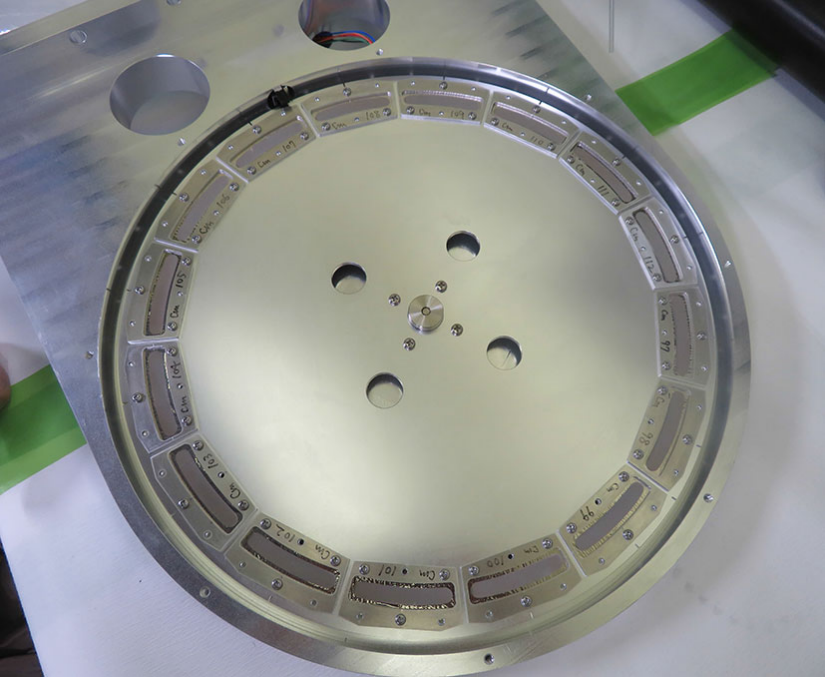
Photograph of the rotating wheel with sixteen $^{248}$Cm sector targets in the semiclosed inner-target box. The cover plate of the inner-target box is removed to display the interior

Historically, at RIKEN, the $^{248}$Cm targets used for the production and decay studies of $^{261}$Rf [63–65], $^{262}$Db [66], $^{265}$Sg [67], $^{266}$Bh [68, 69], and $^{292,293}$Lv [70] included six sectors and were arranged on a rotating (1,000 rpm) wheel with a diameter of 10 cm. The typical beam intensities used were 7, 4, 3, 3, and 0.9 p$\mu$A for $^{18}$O, $^{19}$F, $^{22}$Na, $^{23}$Na, and $^{48}$Ca, respectively. The available $^{248}$ $\hbox {Cm}_2$ $\hbox {O}_3$ material (< 7 mg) at the RNC was used for these GARIS experiments. Based on the insights gained through the GARIS experiments, a $^{248}$Cm target wheel with a large diameter was designed for the Z=119 search experiment. This large target wheel is located in a water-cooled target box in GARIS-II or -III and rotates using a motor at 2,000 rpm in He atmosphere (33−73 Pa) during irradiation. Figure 17 shows the 30-cm diameter target wheel with sixteen sector targets. The semiclosed inner-target box described in subsection 4.1.4 is partly visible.

The $^{248}\hbox {Cm}_2\hbox {O}_3$ target and backing foil need to withstand the exceedingly severe heat produced by the $^{51}$V beam energy loss. Although the severity depends on the experimental conditions, the heat power would, for example, amount to 10 W for a 1 p$\mu$A beam with an energy loss 10 MeV. Because of such high-power, even under high-speed rotation, the temperature of the target/backing foil increases rapidly to 500–1,000$^{\circ }$C depending on the surrounding cooling state, which mainly includes thermal radiation and conduction, rapidly damaging the target/backing foil. Thus, it is necessary and inevitable to develop a target and a backing material that withstand the intense beam and sustain for an extended period, for new SHE experiments. It is risky to use the precious $^{248}$Cm material for such tests. Therefore, at the RNC, $\hbox {Gd}_2\hbox {O}_3$ was utilized instead of $\hbox {Cm}_2\hbox {O}_3$ because both belong to the same chemical family and are expected to show similar behavior. Various backing materials such as Be, C, Ti, or Mo with several different thicknesses are being explored under actual experimental conditions.

## Search for Z = 119 using GARIS-II and GARIS-III

The search for the Z = 119 element through the $^{51}$V$+^{248}$Cm hot fusion reaction commenced in 2018 with GARIS-II for $\sim$1.5 years, and subsequently in 2020 with GARIS-III after the completion of SRILAC.

For full-scale measurement, a considerable quantity of target material ($^{248}\hbox {Cm}_2\hbox {O}_3$) is needed to fill the target wheel as shown in Fig. 17. The required quantity of highly enriched $^{248}$ $\hbox {Cm}_2\hbox {O}_3$ was provided to the RNC under the material transfer agreement between the RNC and ORNL. The supplied $^{248}$Cm material was produced under the U.S. Department of Energy’s isotope program within the $^{252}$Cf production program at ORNL; see [71] for more details. All the GARIS-II and GARIS-III experiments described in this report were performed using the ORNL-made $^{248}\hbox {Cm}_2\hbox {O}_3$ material.

One of the most decisive quantities that influences the yield of a new element through the hot fusion reaction is the bombarding energy. As the theoretical predictive power of the reaction bombarding energy remains premature, it is desirable to presume or obtain it based on experimental results. We first briefly describe our approach for determining the bombarding energy in the following subsection. We then report the current status of the experiments conducted with GARIS-II and GARIS-III.

### Selection of the optimal bombarding energy

The selection of the optimal bombarding energy $(E_{\textrm{opt}})$ is critical.

$E_{\textrm{opt}}$ can be inferred from the fusion barrier distribution, which can be experimentally deduced from the excitation function of the quasielastic (QE) scattering to backward angles. Timmers et al. tested this methodology for the first time [72]. The measurement at $\theta _{\textrm{lab}}\!\sim \! 180^{\circ }$ is significant because the reaction is dominated by the angular momentum $\ell \! \sim \! 0\hbar$, which is a critical component leading to system fusion.

Recently, Tanaka et al. measured the excitation functions of QE backscattering ($\theta _{\textrm{lab}}\!\sim \!180^{\circ }$) using RILAC and GARIS for the $^{22}$Ne$+^{248}$Cm, $^{26}$Mg$+^{248}$Cm, and $^{48}$Ca$+^{238}$U systems [18] and for the $^{48}$Ca$+^{208}$Pb, $^{50}$Ti$+^{208}$Pb, and $^{48}$Ca$+^{248}$Cm systems [19]. They could deduce the fusion barrier distributions for these systems. Note that it was not possible to measure the $^{51}$V$+^{248}$Cm system because RILAC could not provide $^{51}$V energy beyond 5.5 MeV/*u*. However, their systematic data are beneficial because they can be extrapolated to the $^{51}$V$+^{248}$Cm system. The extrapolated value of the average Coulomb barrier height $\hbox {B}_0$ for the $^{51}$V$+^{248}$Cm system was found to be 228.3$\pm 1.1$ MeV. The above $\hbox {B}_0$ value was applied to deduce the bombarding energy in the measurement with GARIS-II in 2018–2019.

Very recently, the barrier distribution for the $^{51}$V$+^{248}$Cm system was obtained by measuring the QE backscattering cross-sections with GARIS-III when SRILAC was operational, by Tanaka et al. [73].

**Fig. 18:**
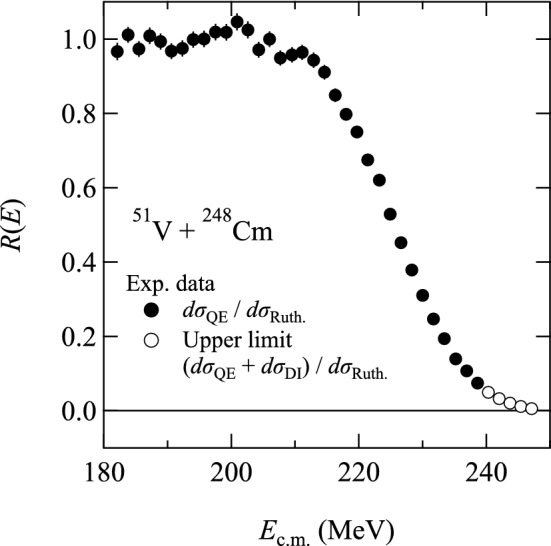
Excitation function of the ratio *R*(*E*) of the QE cross-section to the Rutherford scattering cross-section obtained from Ref. [73]

Figure 18 depicts the ratio *R* as a function of the reaction energy $E_{\mathrm{c.m.}}$ defined by

1 $$\begin{aligned} R = \frac{d \sigma _{\textrm{QE}}}{d\sigma _{\textrm{Ruth}}}, \end{aligned}$$

where $d \sigma _{\textrm{QE}}$ is the QE backscattering cross-section and $d\sigma _{\textrm{Ruth}}$ is the Rutherford scattering cross-section at $\theta _{\textrm{lab}}=180^{\circ }$. $\hbox {B}_0$ is defined as the excitation energy at $R(E)=0.5$.

The deduced $\hbox {B}_0$ value was 225.6 ± 0.2 MeV for the c.m. system; this value is lower by 2.7 MeV than the extrapolated value of 228.3$\pm 1.1$ MeV. According to the $\hbox {B}_0$ value, the bombarding energy was modified in the GARIS-III measurement in 2020.

The final $^{51}$V beam energy $(E_{\textrm{opt}})$ can be determined considering the $\hbox {B}_0$ value, the side collision energy [18, 73], and the thicknesses of the backing material and $^{248}\hbox {Cm}_2\hbox {O}_3$ target material.

Finally, it is important to note that $E_{\textrm{opt}}$ should be achieved at the center of the $^{248}$ $\hbox {Cm}_2$ $\hbox {O}_3$ target material. The typical thickness of the target material is approximately 0.5 mg/cm$^{2}$, corresponding to an energy loss of nearly 4–5 MeV ($\pm 2$ MeV at center). Thus, the accuracy of the beam energy at the center of the target material is required to be better than 0.7%. ($\sim \!\!2$ MeV /300 MeV). This has been sufficiently satisfied in SRILAC, as shown in Sect. 4.1.1.

### Element-119 search with GARIS-II and GARIS-III

The isotopes of new element $^{296}$119 and/or $^{295}$119 can be identified as the evaporation residue implanted in a pixel of the DSSD by observing seven sequential $\alpha$ decays in a chain (seven generations) under an ideal situation. However, such identification becomes problematic when the background particles accidentally enter the same pixel and mimic the expected $\alpha$ decay energy while waiting for a cascading $\alpha$ decay. Thus, a calm environment at the DSSD detector is crucial in terms of the background. In GARIS-II, the background $\alpha$ particle-like accidental events were estimated to be 6.9$\times 10^{-4}/$s at a beam intensity of 2 p$\mu$A for an energy range of 8–15 MeV produced in a 2$\times$4 mm$^2$ pixel of DSSD in the FPD [74], based on the number of events observed in the pixel but not observed in ToF detectors. This low-accidental event rate enables identification of the synthesized element-119 with sufficient certainty under the current experimental conditions.

#### Search with GARIS-II

Irradiation commenced in January 2018 and was completed in May 2019. Here, only the analog data-taking system was employed. The dead-time of a consecutive event was approximately 5 $\mu$s.

Using a 28-GHz ECRIS, RILAC2 along with the RRC provided beam intensities up to 3.5 p$\mu$A for the $^{51}$V$^{13+}$ beam on a $^{248}$Cm target. However, such an intense beam rapidly damages the target/backing material and is therefore, not practically applicable. Hence, we used a significant portion of the available beam time to investigate the lifetime of various backing materials with different thicknesses against an intense $^{51}$V beam, as described in Sect. 6. Despite such efforts, we could not find an appropriate backing material with a suitable thickness. Therefore, finding a better backing material is an urgent necessity, and is being pursued.

During irradiation, the running conditions of all the detectors and target/backing materials were continuously monitored. The accumulated events were analyzed offline by several groups in the nSHE collaboration using an independent analysis program, and the obtained results were compared.

Measurement was performed under irradiation for nearly 80 days in total, and data were obtained for various target backing materials and thicknesses. Careful offline analysis is underway.

#### Search with GARIS-III

As explained in Sect. 7.1, when SRILAC became available, the fusion barrier distribution was first measured with GARIS-III [73]; based on the result, the bombarding energy of the $^{51}$V beam was set for the search of the new superheavy element 119 in GARIS-III.

Since the beginning of this campaign with GARIS-III, special attention has been paid to the target-beam spot shape. The shape of the $^{51}$V beam on the rotating target is carefully adjusted to ensure a uniform distribution of typically 8 mm $\times$1–3 mm in the horizontal and vertical directions, respectively.

**Fig. 19:**
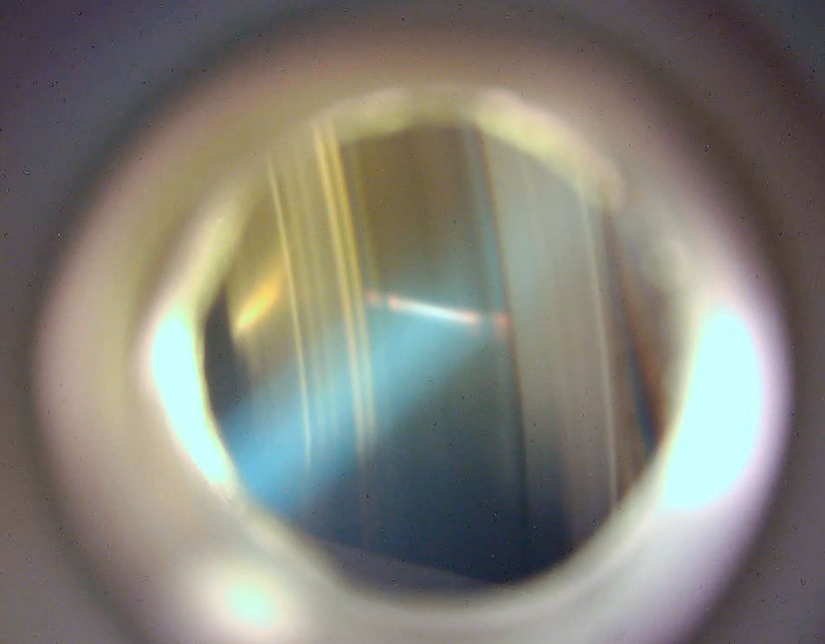
Photograph of the $^{51}$V beam crossing the target area at a high- rotating speed. See text for details

Figure 19 is a snapshot of the target area from the downstream viewing port of the GARIS-III target chamber. See Fig. 10 for the position of the downstream viewing port. A high-intensity $^{51}$V beam from the right-side bombards the rotating $^{248}$Cm targets. The blue-light is due to the luminescence emitted by He-gas along the beam trajectory. The bright spot is due to the $^{248}$Cm target-area transited by the beam, with a size of approximately 8 mm$^{\textrm{H}}\times$1 mm$^{\textrm{V}}$.

In addition to the analog electronics, the digital electronics system was available for this campaign. Thus, the dead-time loss was minimized as mentioned in Sect. 4.2.3.

In the $\sim$1.5 years up to the end of 2021, 95 days were allocated for $^{248}$Cm$+^{51}$V measurement.

## Summary and perspectives

This article described the “SHE project” initiated in 2016 at the RNC. The construction of SRILAC to boost the beam energy from 5.5 MeV/*u* to 6.5 MeV/*u* for rendering hot fusion reaction $^{51}$V$+^{248}$Cm possible to synthesize a new element (119) was one of the main objectives of the project.

During commissioning, which was completed in 2019, the performances of SRILAC and the newly built GARIS-III were confirmed. Thus, the initial objective of the SHE project was successfully achieved in its entirety.

The search for new element Z = 119 was initiated with GARIS-II along with the RILAC2 and RRC accelerators during the SRILAC construction period (2017–2019), and was subsequently continued with the newly built SRILAC and GARIS-III facility.

Unfortunately, as the commissioning and search for a new element were frequently interrupted during 2020 and 2021, the efficiency was below expectation. The various delays caused by the COVID-19 pandemic is an apparent reason. Moreover, a severe breakdown of the 40-year-old linac, RILAC, occurred. It should also be mentioned that the highly efficient $^{248}$Cm material recovery work in the damaged target wheel and the restoration to a new target wheel contributed to a nonnegligible time.

Efforts are being made to accumulate a dose equivalent to $\sim$10 fb [11–13, 15–17], as per the theoretically predicted typical cross-section, as soon as possible.

In principle, the RNC can currently execute two new SHE search experiments simultaneously with the SRILAC +GARIS-III facility and the RILAC2+RRC+GARIS-II facility if sufficient manpower, target material, and beam time are available.

However, the RRC accelerator is extensively used for RIBF experiments. Thus, obtaining beam time for the SHE search experiment with GARIS-II, which requires considerable of beam time, is challenging. Nonetheless, GARIS-II is highly attractive for research that requires relatively short beam time. For example, the first direct measurement of the atomic mass of superheavy nucleus $$^{257}$$Db (Z = 105) was successfully performed through fusion-evaporation reaction $$^{208}$$Pb($$^{51}$$V,$$2n)^{257}$$Db with GARIS-II combined with an apparatus of a multireflection time-of-flight mass spectrograph at the focal plane [75].

In the SRILAC+GARIS-III facility, the available beam time can be utilized by the new element research group for measurement with an intense $^{51}$V beam. This is the right time for accelerating the research to discover element 119, which is the primary objective of the “SHE project.”

## Data Availability

This manuscript has no associated data or the data will not be deposited. [Authors’ comment: The data presented in this study are available from the corresponding author on reasonable request.]

## Footnotes

1 “n” could denote “n”ew, “n”ishina, or “n”ihon.

2 1 p$\mu$A is equal to $6.24 \times 10^{12}$ particles/s.

## References

[1] For example, https://www.nishina.riken.jp/about/history_e.html

[2] Odera M (1984). Nucl. Instrum. Methods.

[3] Morita K (2004). J. Phys. Soc. Jpn..

[4] Morita K (2007). J. Phys. Soc. Jpn..

[5] Morita K (2012). J. Phys. Soc. Jpn..

[6] Morita K (2009). J. Phys. Soc. Jpn..

[7] Morita K (2004). Eur. Phys. J. A.

[8] Oganessian YT (2006). Phys. Rev. C.

[9] Karol PJ (2016). Pure Appl. Chem..

[10] Hofmann S, Dimitriev SN, Fahlander C, Gates JM, Roberto JB, Sakai H (2020). Pure Appl. Chem..

[11] Ghahramany N, Ansari A (2016). Eur. Phys. J. A.

[12] Zhu L, Xie WJ, Zhang FS (2014). Phys. Rev. C.

[13] Adamian GG, Antonenko NV, Lenske H (2018). Nucl. Phys. A.

[14] Manjunatha HC, Sridhar KN, Ramalingam HB (2019). Nucl. Phys. A.

[15] Siwek-Wilczynska K, Cap T, Kowal M (2019). Phys. Rev. C.

[16] Aritomo Y (2020). JPS Conf. Proc..

[17] Lv X-J, Yue Z-Y, Zhao W-J, Wang B (2021). Phys. Rev. C.

[18] Tanaka T (2020). Phys. Rev. Lett..

[19] Tanaka T (2018). J. Phys. Soc. Jpn..

[20] Kaji D, Morimoto K, Sato N, Yoneda A, Morita K (2013). Nucl. Instrum. Meth. B.

[21] Okuno H (2012). Prog. Theor. Exp. Phys..

[22] Kamigaito O (2005). Rev. Sci. Instrum..

[23] O. Kamigaito et al., Proc. IPAC2016, Busan, May 2016, TUPMR022, (2016) p. 1281

[24] Nagatomo T (2020). Rev. Sci. Instrum..

[25] Nakagawa T (2010). Rev. Sci. Instrum..

[26] Higurashi Y (2012). Rev. Sci. Instrum..

[27] Suda K (2013). Nuc. Instrum. Methods.

[28] J. Wei et al., Proc. LINAC2016 Conf., East Lansing, MI, USA, Sep. 2016, paper MOA01, p. 1

[29] R. Ferdinand et al., Proc. Linac16 Conf., East Lansing, MI, USA, Sep. 2016, paper WE1A06, p. 668

[30] D. Jeon et al., Proc. SRF2017 Conf., Lanzhou, China, Jul. 2015, paper MOYA03, p. 36

[31] Yang JC (2013). Nucl. Instrum. Methods Phys. Res. B.

[32] Barth W (2018). Phys. Rev. Acc. Beams.

[33] K. Yamada et al., Proc. SRF2013, Paris, MOP021, p. 137 (2013)

[34] N. Sakamoto et al., Proc. SRF2015, Whistler, WEBA06, p. 976 (2015)

[35] N. Sakamoto et al., Proc. LINAC2018, Beijing, China, paper WE2A03, p. 620

[36] Ostroumov PN, Shepard KW (2001). Phys. Rev. ST. Accel. Beams.

[37] T. Watanabe et al., Proc. IBIC2019, Malmö, Sweden, paper WEPP007, p. 526

[38] H. Imao et al., Proc. SRF2019, Dresden, Germany, paper TUP013, p. 419

[39] Alton GD, Smithe DN (1994). Rev. Sci. Instrum..

[40] Higurashi Y (2014). Rev. Sci. Instrum..

[41] J. Ohnishi et al., Proc. ECRIS2018, Catania, Italy, paper WEB4, p. 180

[42] Yamada K (2019). RIKEN Accel. Prog. Rep..

[43] S. Kimura, D. Kaji et al., in preparation

[44] Kaji D (2013). Nucl. Instrum. Methods B.

[45] Kaji D (2015). J. Radioanal. Nucl. Chem..

[46] Kaji D (2017). J. Phys. Soc. Jpn..

[47] Imao H (2019). RIKEN Accel. Progr. Rep..

[48] Ishizawa S, Morimoto K, Kaji D, Tanaka T, Tokanai F (2020). Nucl. Instrum. Methods A.

[49] CREMAT https://www.cremat.com/home/charge-sensitive-preamplifiers/

[50] John Bland C (1998). App. Radiat. Isot..

[51] P. Brionnet et al., submitted to Nucl. Instrum. Methods A

[52] Pixie-16. https://xia.com/support/pixie-16/

[53] Vermeulen D, Clerc H-G, Sahm C-C (1984). Z. Phys. A.

[54] P. Brionnet et al., nSHE internal report. To be published

[55] F.P. He$$\beta $$berger, EPJ D **45**, 33 (2007)

[56] http://nrv.jinr.ru/nrv/

[57] Reisdorf W, Schädel M (1992). Z. Phys. A.

[58] K. Morimoto, Invited talk at 13th International Conference on Nucleus-Nucleus Collisions, December 4-8, 2018 held in Omiya, Japan

[59] Nakagawa T, Yano Y (2000). Rev. Sci. Instrum..

[60] Rubert J, Piot J, Asfari Z, Gall B (2012). Nucl. Instrum. Methods B.

[61] B. Gall et al., in preparation

[62] Kudou Y (2009). RIKEN Accel. Progr. Rep..

[63] Haba H (2009). Chem. Lett..

[64] Haba H (2011). Phys. Rev. C.

[65] Murakami M (2013). Phys. Rev. C.

[66] Haba H (2014). Phys. Rev. C.

[67] Haba H (2012). Phys. Rev. C.

[68] Morita K (2009). J. Phys. Soc. Jpn..

[69] Haba H (2020). Phys. Rev. C.

[70] Kaji D (2017). J. Phys. Soc. Jpn..

[71] J.B. Roberto et al., Actinide targets for the synthesis of superheavy nuclei, in this volume

[72] Timmers H (1995). Nucl. Phys. A.

[73] Tanaka M (2022). J. Phys. Soc. Jpn..

[74] S. Ishizawa, PhD thesis, Yamagata University (2021)

[75] Schury P (2021). Phys. Rev. C.

